# Putative Auxin and Light Responsive Promoter Elements From the *Tomato spotted wilt tospovirus* Genome, When Expressed as cDNA, Are Functional in *Arabidopsis*

**DOI:** 10.3389/fpls.2019.00804

**Published:** 2019-06-28

**Authors:** Ying Zhai, Hao Peng, Michael M. Neff, Hanu R. Pappu

**Affiliations:** ^1^Department of Plant Pathology, Washington State University, Pullman, WA, United States; ^2^Department of Crop and Soil Sciences, Washington State University, Pullman, WA, United States

**Keywords:** auxin, CCA1, light, promoter, RNA virus, TSWV, untranslated region

## Abstract

Members of the virus order *Bunyavirales* cause serious diseases in animals, humans and plants. Family *Tospoviridae* in this order contains only one genus *Orthotospovirus*, and members in this genus exclusively infect plants. *Tomato spotted wilt tospovirus* (TSWV) is considered one of the most economically important plants viruses. Little is known about the regulatory elements in the TSWV genome. Here we show that, when in the cDNA form, the 5′-upstream region of the TSWV-coded G_N_/G_C_ gene (pG_N_/G_C_) possesses putative *cis*-regulatory elements, including an auxin responsive element (AuxRE) for binding of auxin response factors (ARFs), as well as a circadian clock-associated 1 (CCA1) protein binding site (CBS). Due to the lack of a reverse genetics system, we verified the functionality of these elements in Arabidopsis. pG_N_/G_C_ showed light-suppressive promoter activity in transgenic Arabidopsis, and mutation in the CBS was sufficient to switch the activity to light inducible. Additionally, exogenous auxin treatments repressed the promoter activity of both wild type and CBS-mutated pG_N_/G_C_. Mutation in AuxRE in both promoters abolished their sensitivity to auxin. As transcriptional repressors, both CCA1 and ARF2 were able to bind to pG_N_/G_C_ directly. To our knowledge, this is the first report that a 5′-terminal sequence of an RNA virus has light-and hormone-responsive promoter activities when expressed as cDNA in host plant’s nuclear background. Our findings suggest new clues on the possible origin, evolution and function of the TSWV genomic sequence and its non-coding regions.

## Introduction

Viruses in the order *Bunyavirales* are characterized by segmented RNA genome with three RNAs packaged in enveloped virus particles (Briese et al., 2013). Family *Tospoviridae* contains only one genus *Orthotospovirus* (Siddell et al., 2019). Viruses in this genus exclusively infect plants, and tospoviruses are unique in that the large RNA is in negative sense while the medium and small RNAs possess an ambisense genome organization (Prins and Goldbach, 1998; Adkins, 2000). T*omato spotted wilt tospovirus* (TSWV), a member of the genus *Orthotospovirus*, is considered one of the world’s most important plant viruses (Scholthof et al., 2011). Transmitted by thrips, TSWV causes significant losses to a wide range of economically important crops (Pappu et al., 2009; Oliver and Whitfield, 2016). The three genomic, single-stranded RNAs encode all the essential proteins for virus infection, replication, and particle assembly (Zhai et al., 2014; Tripathi et al., 2015). In recent years, considerable progress has been made on elucidating the tospovirus genome organization, replication, transcription, and molecular interactions with its host plants (Turina et al., 2016).

RNA viral genomes encode a RNA-dependent RNA polymerase (RdRp) that recognizes specific RNA elements (promoter sequences) in either the positive (+) or negative (-) strands of the viral RNA genomes, and viral *cis*-regulatory elements (either outside or inside the coding regions) may play an important role in virus replication and RdRp-RNA interactions (Koev and Miller, 2000; Li and Stollar, 2007). During tospoviral transcription, the viral RdRp does not add a cap structure to mRNAs due to its lack of methyltransferase activity. Instead, the tospoviral RdRp snatches capped RNA leader sequences from the cytoplasmic pool of host mRNAs and uses these sequences to prime transcription (Kormelink et al., 1992; Duijsings et al., 2001; van Knippenberg et al., 2005). Both tospovirus replication and transcription take place entirely in the cytoplasm, but little is known about the host factors involved or the source of capped RNA leader sequences (Turina et al., 2016).

A major bottleneck in studying structure-function relationships of tospoviruses is the lack of a reverse genetics system. The ambisense genome organization of tospoviruses makes it very difficult to produce an infectious cDNA clone of the RNA genome. Despite years of effort by several groups, an infectious clone for any tospovirus remains elusive. Additionally, the roles of non-translated terminal regions of the genomic RNAs in tospovirus gene expression are largely unknown.

Here we show that, when in the cDNA form, the 5′-upstream region of the TSWV-coded G_N_/G_C_ gene (pG_N_/G_C_) possesses putative *cis*-regulatory elements, including an auxin response element (AuxRE) for binding of auxin response factors (ARFs) (Guilfoyle and Hagen, 2007), as well as a circadian clock-associated 1 (CCA1) protein binding site (CBS) (Wang and Tobin, 1998). Since there is no reverse genetic system available for any known tospovirus, we verified the functionality of these elements in Arabidopsis. To our knowledge, this is the first report of a 5′-terminal sequence of an RNA virus with light-and hormone-responsive promoter activities when expressed as cDNA in a plant host.

## Materials and Methods

### Plant, Bacteria, Virus, and Plasmid Materials

*Arabidopsis thaliana* ecotype *Col*-0 was used for *Agrobacterium*-mediated transformation. The *Agrobacterium tumefaciens* strain GV3101 was used for *Arabidopsis* transformation. The TSWV isolate T (M segment GenBank no. AY870389) was used. pCAMBIA1381Z was used as the plant expression binary vector.

### Promoter Analysis of *cis*-Regulatory Elements

The 5′-upstream sequences of the TSWV-coded genes were scanned for putative plant *cis*-regulatory elements. The list of known transcription factor (TF)-binding sites in plants was obtained from the Arabidopsis *cis*-regulatory element database (AtcisDB) in the Arabidopsis Gene Regulatory Information Server (AGRIS)^1^. Except for the transcription initiation site, both the original and the reverse-complement sequences of the TF-binding sites were used for scanning.

### RNA Extraction, Reverse Transcription and DNA Cloning

Total RNA was extracted from TSWV infected *Nicotiana benthamiana* leaves for cDNA synthesis. The pG_N_/G_C_ fragment was amplified from cDNA using primers 5′-CGGAATTCAGAGCAATCAGTGCAAACAAA -3′ and 5′- CGGGATCCTTATTTTCCACTTGATAATAAACATTA -3′, and then cloned into *Eco*RI/*Bam*HI sites of pCAMBIA1381Z. The pN fragment was amplified using primers 5′- CGGAATTCAGAGCAATCGTGTCAATTTTGTGTT -3′ and 5′- CGGGATCCGTATTGAGATTCTCAGAATTCCC -3′, and then cloned into *Eco*RI/*Bam*HI sites of pCAMBIA1381Z. The pRdRp fragment was amplified using primers 5′- CGGAATTCAGAGCAATCAGGTAACAACGA -3′ and 5′- CGGGATCCTTATTTATTCTCTCAAACTCATCATC -3′, and then cloned into *Eco*RI/*Bam*HI sites of pCAMBIA1381Z. All the PCR amplicons were verified by sequencing.

### Plant Transformation and Seedling Growth

The constructs were introduced into *Agrobacterium* by electroporation and then used to transform *Arabidopsis* using the floral dip method (Clough and Bent, 1998). T_2_ seeds were collected from T_1_ transgenic plants and used for the selection of single-locus insertion transgenic lines (3:1 segregation of hygromycin resistant versus sensitive seedlings, verified by the chi-square test). Homozygous transgenic seeds were obtained in the T_3_ generation. Two independent transgenic lines with single-locus T-DNA insertions were used for every promoter activity and GUS staining assay. To prepare material for PCR and GUS assays, seeds were placed on half-strength MS medium plates. After stratification at 4°C for 4 days, the seeds were treated by red light for 5 h to stimulate germination, followed by growing at 25°C for 4 days, either in dark or under continuous 80 μmol m^-2^ s^-1^ white light (Peng et al., 2015). For indole-3-acetic acid (IAA) treatments, seeds were placed on IAA-containing medium plates throughout the experiments.

### Quantitative RT-PCR and GUS Staining

Total RNA was extracted from 4-day old seedlings (grown in either in dark or under light), Sigma RNase-free DNase I (St. Louis, MO, United States) was added during the RNA extraction to reduce gDNA contamination. Reverse transcription was done using the iScript Reverse Transcription Supermix (Bio-Rad, Hercules, CA, United States). Quantitative RT-PCR (qRT-PCR) was performed using the SsoAdvanced Universal SYBR Green Supermix (Bio-Rad) and the Applied Biosystems 7500 fast real-time PCR system (Grand Island, NY, United States). The qRT-PCR primers for the detection of *GUS* transcripts were 5′-GGTAGATCTGAGGAACCGACG-3′ and 5′-TCGCGATCCAGACTGAATGCC-3′. The forward primer stretches over the catalase intron of the *GUS* gene (13 nt upstream and 8 nt downstream of the catalase intron). The *Arabidopsis UBQ10* gene was used as reference. The qRT-PCR primers for the detection of *UBQ10* transcripts were 5′-TCTTCGTGGTGGTTTCTAAATCTCG-3′ and 5′-AAAGAGATAACAGGAACGGAAACATAGT-3′. Each data point had three biological replicates. Unpaired Student’s *t*-test was used to test the significance of difference in gene expression. The GUS staining was performed using the protocol previously described (Jefferson et al., 1987).

### Targeted Yeast One-Hybrid Assay

The Gateway-compatible system was adopted for the Y1H assay (Deplancke et al., 2006). The DNA fragment (baits, pG_N_/G_C_-CBS in this case) was cloned into pDONR P4-P1R by BP reaction. The fragment was then subcloned into pMW#2 by LR reaction and integrated into the genome of yeast strain YM4271. pG_N_/G_C_-CBS was fused with the reporter gene *HIS3* in the yeast genome. After verifying for self-activation, the bait yeast strain was transformed with *CCA1* or *ARF2* cloned in the Gateway prey vector pACT-GW and the empty vector as control. The activation of *HIS3* was tested by yeast tolerance to 3-aminotriazole (3-AT, a competitive inhibitor of the His3p enzyme). The primers used for the initial amplification of pG_N_/G_C_-CBS were 5′- GGGGACAACTTTGTATAGAAAAGTTGGACTAATCTGATGCTAGAATCTC-3′ and 5′- GGGGACTGCTTTTTTGTACAAACTTGGAAGCATTCAAGCAGTTGTTAGG-3′. The sequence of pG_N_/G_C_-CBS is GACTAATCTGATGCTAGAATCTCAGACTCCTGGAACCCGTCAGATACGAGAAGAAGAATCAACCATCCCTATTTTTGCTGAGTCAACTACGGAAAAAACAATCTTTGTCTCGGATCTTCCTAACAACTGCTTGAATGCTTC. All the PCR amplicons were verified by sequencing. All the Gateway cloning reagents came from Invitrogen.

### Site-Directed Mutagenesis

The QuickChange site-directed mutagenesis kit (Agilent Technologies) was used for mutating the CBS and AuxRE elements in the pG_N_/G_C_:*GUS* construct. The primers used to mutate CBS (AACAATCT) to CBSm (AACGGTCT) are 5′-CTGAGTCAACTACGGAAAAAACGGTCTTTGTCTCGGATCTTCCTAA-3′ and 5′-TTAGGAAGATCCGAGACAAAGACCGTTTTTTCCGTAGTTGACTCAG-3′. The primers used to mutate AuxRE (TGTCTC) to AuxREm (TGGATC) are 5′-AAGCAGTTGTTAGGAAGATCCGATCCAAAGATTGTTTTTTCCGTAGTTG-3′ and 5′-CAACTACGGAAAAAACAATCTTTGGATCGGATCTTCCTAACAACTGCTT-3′. The primers used to mutate CBS and AuxRE simultaneously are 5′-CAGTTGTTAGGAAGATCCGATCCAAAGACCGTTTTTTCCGTAG-3′ and 5′-CTACGGAAAAAACGGTCTTTGGATCGGATCTTCCTAACAACTG-3′. All the introduced mutations were verified by sequencing.

## Results

### Sequence Analysis of the 5′-Upstream Regions of Five TSWV cDNAs

**FIGURE 1:**
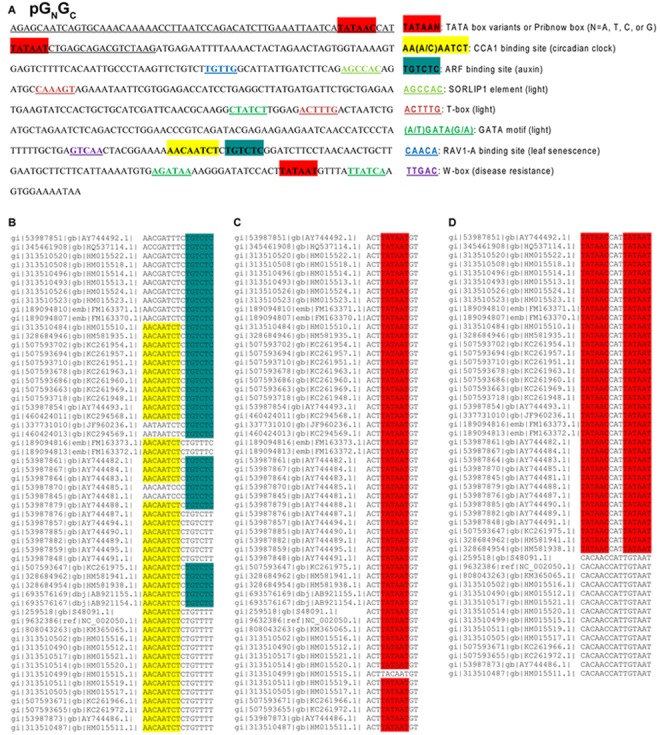
The *cis*-regulatory elements identified from the 5′-upstream 500-bp region of *Tomato spotted wilt tospovirus* (TSWV) gene (cDNA) G_N_/G_C_ (named as pG_N_/G_C_) and the conservatism of putative CBS, AuxRE and transcription initiation sites in pG_N_/G_C_. **(A)** The 5′-upstream 500-bp region includes both non-translational sequence and the 5′-terminal gene sequences. The underlined sequence represents untranslated region. pG_N_/G_C_ has putative transcription initiation sites and light-responsive transcription factor binding elements. It also has putative CBS and AuxRE for the binding of CCA1 and ARFs, respectively. **(B)** Alignment of 56 TSWV G_N_/G_C_ sequences available in GenBank showed that at least one CBS or AuxRE element can be identified in pG_N_/G_C_ of any given TSWV isolate. **(C)** The Pribnow box downstream of CBS and AuxRE is present in 55 out of 56 TSWV isolates examined. **(D)** The upstream TATA and Pribnow boxes in the 5′-untranslated region can only be found in 36 out of 50 TSWV isolates with complete sequences available.

Whether TSWV non-coding regions have regulatory function in virus replication or gene expression is still unknown. Due to the lack of a reverse genetics system for tospoviruses, it is difficult to investigate the potential functions of these non-coding regions in their original RNA form. Hence we started the functional dissection of the TSWV non-coding regions in their respective cDNA forms. All three genomic cDNAs of TSWV isolate T were scanned for potential transcription initiation and transcription factor (TF) binding elements. As it is hard to define the precise boundaries for promoters, we analyzed the 500 bp in the 5′-upstream regions (including both non-translational sequences and the 5′-terminal gene sequences) of the five TSWV genes. No putative transcript initiation sites (TATA box or its variants) were found in the 5′-upstream regions of RdRp, NSm and NSs (named as pRdRp, pNSm, and pNSs, respectively, Supplementary Figures S1A–C). However, both pG_N_/G_C_ (Figure 1A) and the 500-bp fragment upstream of the N gene (named pN; Supplementary Figure S1D) were found to contain a putative TATA box variant (TATAAC) and Pribnow box (TATAAT). The promoter activity of TATAAC was predicted to be weaker than the standard TATA box (TATAAA) (Narang et al., 2005). The Pribnow box is essential for transcription in bacteria but may also have some promoter activity in eukaryotes (Patikoglou et al., 1999). Three putative light-responsive TF-binding elements were found in both pG_N_/G_C_ (Figure 1A) and pN (Supplementary Figure S1D), including a SORLIP1 element (Hudson and Quail, 2003), a T-box motif (Chan et al., 2001), and a GATA binding motif (Teakle et al., 2002). pG_N_/G_C_ was also found to contain CBS and AuxRE (Figure 1A), which are not present in pN (Supplementary Figure S1D).

### The Conservation of Putative Promoter Elements in pG_N_/G_C_

Alignment of 56 TSWV G_N_/G_C_ sequences available in GenBank showed that at least one CBS or AuxRE is present in pG_N_/G_C_ of any given TSWV isolate (Figure 1B). 20 out of 56 isolates contained both elements, 22 isolates have only CBS and 14 isolates have only AuxRE (Figure 1B). With the exception of TSWV, neither CBS nor AuxRE was found in the corresponding same region of other known tospoviral genomes. The Pribnow box, downstream of CBS and AuxRE, is present in 55 out of 56 isolates examined (Figure 1C), while the upstream TATA and Pribnow boxes in the 5′-untranslated region were found in 36 of the 50 isolates for which complete sequences are available (Figure 1D).

### pG_N_/G_C_ Has Light-Suppressive Promoter Activity

**FIGURE 2:**
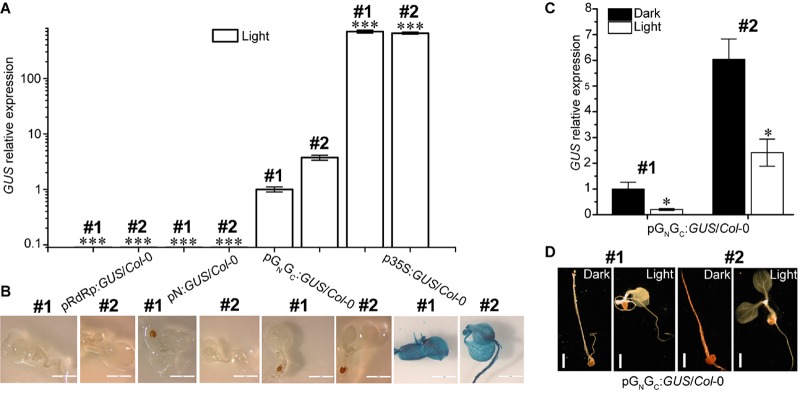
pG_N_/G_C_ of the medium RNA (cDNA) of TSWV has light-suppressive promoter activity. **(A)** qRT-PCR assay using two independent transgenic lines (#1 and #2) for each genotype revealed that pG_N_/G_C_ can drive the expression of a downstream *GUS* gene. pG_N_/G_C_ showed dramatically stronger promoter activity than both pN and pRdRp, but was weaker than p35S. **(B)** GUS protein accumulations can only be detected in p35S:*GUS*/*Col*-0 seedlings. **(C)** qRT-PCR assay using two independent transgenic lines (#1 and #2) showed that the promoter activity of pG_N_/G_C_ was down regulated by light. **(D)** There was no detectable GUS protein accumulations in pG_N_/G_C_:*GUS*/*Col*-0 seedlings (#1 and #2) either in dark or in light. All transgenic Arabidopsis plants (pG_N_/G_C_:*GUS*/*Col*-0 and pN:*GUS*/*Col*-0) had single-locus T-DNA insertions. Two independent transgenic lines (#1 and #2) were used for each qRT-PCR and GUS staining assay. In **(A,B)**, seedlings were grown for 4 days under 80 μmol m^-2^ s^-1^ continuous white light. In **(C,D)**, seedlings were grown for 4 days in dark and 80 μmol m^-2^ s^-1^ white light conditions, respectively. For qRT-PCR assays, three biological replicates were conducted for each data point. The error bar denotes SEM. Stars indicate significant difference when tested by unpaired Student’s *t*-test (^∗^*P* < 0.05, ^∗∗∗^*P* < 0.001). In **(A)**, comparisons of *GUS* expression levels were made between the dedicated groups and the groups of pG_N_/G_C_:*GUS*/*Col*-0. In (**C**), comparisons of *GUS* expression levels were made between dark- and light-grown groups of pG_N_/G_C_:*GUS*/*Col*-0. For seedling images in **(B)**, scale bar = 2 mm. For seedling images in **(D)**, scale bar = 1 mm.

Both pG_N_/G_C_ and pN contain a putative TATA box variant and therefore may have promoter activity (Bernard et al., 2010). To test this hypothesis, the pG_N_/G_C_ and pN fragments were cloned into the binary vector pCAMBIA1381Z to make promoter-*GUS* fusions (pG_N_/G_C_:*GUS* and pN:*GUS*), respectively. The TATA-absent pRdRp fragment was also cloned into pCAMBIA1381Z (pRdRp:*GUS*) for use as a negative control. GUS driven by the constitutive CaMV 35S promoter (p35S:*GUS*) was used as the positive control. As a compatible host for TSWV, Arabidopsis ecotype *Col*-0 was used for pG_N_/G_C_:*GUS* transformation (German et al., 1995). Homozygous transgenic lines with single-locus T-DNA insertion were selected for successive experiments. Four-day old seedlings were used for assessing the promoter activity. Quantitative RT-PCR (qRT-PCR) was used to detect *GUS* transcript accumulation levels in seedlings grown under continuous white light (80 μmol m^-2^ s^-1^). Two independent transgenic lines were tested for each construct. Same transgenic plant selection criteria, seedling age and growth conditions applied to all the assays in this research. The promoter strengths of both pRdRp (negative control) and pN were negligible when compared to that of pG_N_/G_C_ (Figure 2A), which demonstrated that pG_N_/G_C_ can drive the transcription of the downstream *GUS* gene. In contrast, pN may not have meaningful promoter activity since pN:*GUS*/*Col*-0 lines only had basal-level *GUS* expression similar to that of pRdRp:*GUS*/*Col*-0 lines (Figure 2A). Compared to CaMV 35S, pG_N_/G_C_ showed much weaker promoter activity (Figure 2A). While p35S:*GUS* lines showed strong GUS signal as expected, no visible GUS staining was detected from pRdRp:*GUS*, pN:*GUS* or pG_N_/G_C_:*GUS* transgenic lines (Figure 2B). Furthermore, the activity of pG_N_/G_C_ was down regulated by white light treatment when comparing GUS expression levels in dark- and light-grown pG_N_/G_C_:*GUS*/*Col*-0 seedlings (Figure 2C). In contrast, white light versus dark did not significantly affect the expression of p35S:*GUS* (Supplementary Figure S2). No visible GUS signal was observed in either light- or dark-grown pG_N_/G_C_:*GUS*/*Col*-0 seedlings (Figure 2D). It could be that either that the accumulated GUS protein was not sufficient to generate a visible signal, or the translated GUS protein was degraded due to it being targeted to an inappropriate location in the plant cell. Since part of the G_N_/G_C_ gene sequence was fused to the *GUS* gene, it may also have affected the stability of the GUS protein.

### CCA1 Binds to the CBS Element on pG_N_/G_C_

**FIGURE 3:**
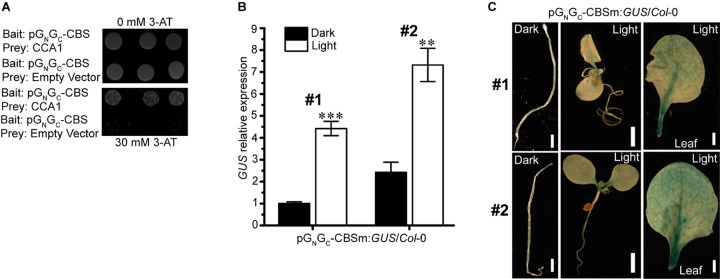
The light-suppressive promoter activity of pG_N_/G_C_ of the medium RNA (cDNA) of *Tomato spotted wilt tospovirus* is regulated by the circadian clock protein CCA1. **(A)** CCA1 interacted with pG_N_/G_C_-CBS in targeted Y1H assay. **(B)** Disruption of CBS on pG_N_/G_C_ switches its promoter activity to light-inductive. qRT-PCR assay using two independent transgenic lines (#1 and #2) revealed that the promoter activity of CBS-mutated pG_N_/G_C_-CBSm was up-regulated by light. **(C)** GUS staining results indicated that more GUS protein accumulated in light-grown pG_N_/G_C_-CBSm:*GUS*/*Col*-0 seedlings (#1 and #2) than in the dark-grown counterpart. Three independent yeast clones were shown for each sample. All transgenic Arabidopsis plants (pG_N_/G_C_-CBSm:*GUS*/*Col*-0) have single-locus T-DNA insertions. Two independent transgenic lines (#1 and #2) were used for each qRT-PCR and GUS staining assay. Seedlings were grown for 4 days at dark or 80 μmol m^-2^ s^-1^ white light condition. Leave samples for GUS staining came from 2-week old plants. For qRT-PCR, three biological replicates were conducted for each data point. The error bar denotes SEM. Stars indicate significant difference (tested by unpaired Student’s *t*-test) of *GUS* expression levels between dark- and light-grown pG_N_/G_C_-CBSm:*GUS*/*Col*-0 seedlings (^∗∗^*P* < 0.01, ^∗∗∗^*P* < 0.001). For seedling and leaf images, scale bar = 1 mm.

Interactions between light-responsive TF-binding elements and their acting TFs may positively or negatively regulate the transcription of downstream genes. The SORLIP1 element, the T-box motif and the GATA binding motif are all light-activating elements (Chan et al., 2001; Teakle et al., 2002; Rus Alvarez-Canterbury et al., 2014), while CCA1-CBS interaction can either activate (Fujiwara et al., 2008) or suppress (Wang and Tobin, 1998; Li et al., 2011) the expression of target genes. Of the four elements above, CBS is the only one that can act as a *cis*-repressor. Since pG_N_/G_C_:*GUS* expression was suppressed by light (Figure 2A), CBS could be the major and *bona fide cis*-regulatory element in pG_N_/G_C_ that mediates light-induced reduction of target gene expression. Targeted yeast one-hybrid (Y1H) assay showed that CCA1 directly bound pG_N_/G_C_ (Figure 3A). A 141-bp CBS-containing sequence from pG_N_/G_C_ (named as pG_N_/G_C_-CBS) was used as the bait in the assay. CBS was the only light-responsive element located in pG_N_/G_C_-CBS.

### Disruption of the CBS Element in pG_N_/G_C_ Switches Its Promoter Activity to Light Inducible

pG_N_/G_C_:*GUS* with CBS mutated from AACAATCT to AACGGTCT (named as pG_N_/G_C_-CBSm:*GUS*) was used for stable transformation of Arabidopsis. Homozygous lines with single-locus T-DNA insertions were selected for subsequent experiments. qRT-PCR analysis of two independent transgenic lines (pG_N_/G_C_-CBSm:*GUS*/*Col*-0 -1 and pG_N_/G_C_-CBSm:*GUS*/*Col*-0 -2) showed that the promoter activity of pG_N_/G_C_-CBSm became light-inducible (Figure 3B). The result suggested that CBS was responsible for the light-suppressive characteristic of pG_N_/G_C_, and its suppression effect was epistasis to other light-inducible elements. The SORLIP1, T-box and GATA elements may contribute to the light-inducible feature of pG_N_/G_C_-CBSm after the disruption of CBS. Consistent with the qRT-PCR result, light-grown pG_N_/G_C_-CBSm:*GUS*/*Col*-0 seedlings and leaves had much stronger GUS staining compared to the leaves from seedlings grown in darkness (Figure 3C).

### Auxin Suppresses the Promoter Activity of pG_N_/G_C_ via ARF-AuxRE Interaction

**FIGURE 4:**
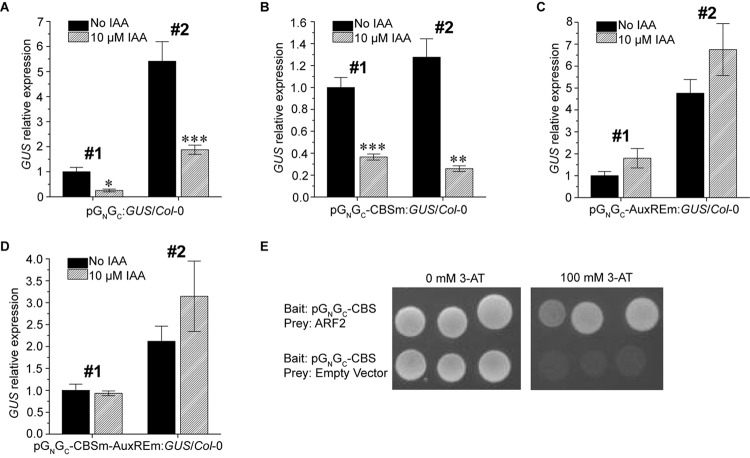
Auxin suppresses the promoter activity of pG_N_/G_C_ of the medium RNA (cDNA) of *Tomato spotted wilt tospovirus* via ARF-AuxRE interaction. The promoter activity of both pG_N_/G_C_ **(A)** and pG_N_/G_C_-CBSm **(B)** was suppressed by exogenous IAA treatments. Disruption of AuxRE (AuxREm) can abolish the suppression effect of exogenous IAA on both pG_N_/G_C_ **(C)** and pG_N_/G_C_-CBSm **(D)**. **(E)** ARF2 directly bind the AuxRE-containing pG_N_/G_C_-CBS fragment in targeted Y1H assay. Three independent yeast clones were shown for each sample. All transgenic Arabidopsis plants (pG_N_/G_C_:*GUS*/*Col*-0, pG_N_/G_C_-CBSm:*GUS*/*Col*-0, pG_N_/G_C_-AuxREm:*GUS*/*Col*-0 and pG_N_/G_C_-CBSm-AuxREm:*GUS*/*Col*-0) have single-locus T-DNA insertions. Two independent transgenic lines (#1 and #2) were used for each qRT-PCR assay. Seedlings were grown for 4 days under 80 μmol m^-2^ s^-1^ white light. For qRT-PCR, three biological replicates were conducted for each data point. The error bar denotes SEM. Stars indicate significant difference (tested by unpaired Student’s *t*-test) of *GUS* expression levels between seedlings treated with 10 μM IAA and non-IAA treatment control groups (^∗^*P* < 0.05, ^∗∗^*P* < 0.01, ^∗∗∗^*P* < 0.001).

Since a putative AuxRE is present in pG_N_/G_C_, the auxin responsiveness of pG_N_/G_C_ was tested using two pG_N_/G_C_:*GUS*/*Col*-0 lines and two pG_N_/G_C_-CBSm:*GUS*/*Col*-0 lines mentioned above. The promoter activities of both pG_N_/G_C_ (Figure 4A) and pG_N_/G_C_-CBSm (Figure 4B) could be suppressed by exogenous indole-3-acetic acid (IAA) treatments. In contrast, IAA did not significantly affect the expression of p35S:*GUS* (Supplementary Figure S2). Similar site-mutagenesis approach was used to mutate the AuxRE from TGTCTC to TGGATC in pG_N_/G_C_:*GUS* and the new construct was named as pG_N_/G_C_-AuxREm:*GUS*. Alternatively, CBS and AuxRE were mutated simultaneously in pG_N_/G_C_:*GUS* to make another new construct named as pG_N_/G_C_-CBSm-AuxREm:*GUS*. Both constructs were used for Arabidopsis transformation. Two independent homozygous transgenic lines with single-locus T-DNA insertion from each transformation event were used for the IAA treatment assay. Seedlings were grown for 4 days under 80 μmol m^-2^ s^-1^ white light. The results showed that disruption of AuxRE (AuxREm) can abolish the suppression effect of exogenous IAA on both pG_N_/G_C_ (Figure 4C) and pG_N_/G_C_-CBSm (Figure 4D). Additionally, in a targeted Y1H assay, the transcriptional repressor ARF2 bound pG_N_/G_C_-CBS, which contains the AuxRE of pG_N_/G_C_ (Figure 4E).

## Discussion

Our results suggest that the TSWV has regulatory elements that are found to be responsive to light and auxin when expressed as cDNA in the plant’s nuclear background. Following our finding that certain promoter-like elements are present in the cDNA of TSWV M RNA, we used Arabidopsis to test if these elements might be functional in a plant host. Our results showed that the putative promoter elements we identified, indeed, were light and auxin responsive when expressed as cDNA in the host nuclear background. A mutation in CBS was sufficient to switch the pG_N_/G_C_ activity from light-suppressive to inducible demonstrating the functional validity of pG_N_/G_C_ in the host plant. Similarly, the promoter activity of pG_N_/G_C_ was no longer suppressed by auxin when AuxRE was mutated. It will be interesting to investigate which element (SORLIP1, T-box or GATA) is the principal contributor to the light inducible characteristic of pG_N_/G_C_-CBSm. It is also possible that these elements simply have additive effect on light response.

Since TSWV is an RNA virus that completes its life cycle in the cell cytoplasm, the pG_N_/G_C_ fragment exists in the native TSWV genome as RNA, and thus cannot act as a traditional promoter for the initiation of transcription. Our unpublished data also indicated that no DNA form of pG_N_/G_C_ could be amplified by PCR from TSWV-infected plant tissues. The replication process of TSWV is not fully understood. It could be that the putative *cis*-regulatory elements in the M RNA may regulate the replication of M RNA or the expression of G_N_/G_C_ mediated by TSWV RdRp. Unlike ARFs which are localized in the nucleus, CCA1 can be detected in both nucleus and cytoplasm (Yakir et al., 2009). Therefore, the CCA1-CBS interaction may also be involved in the replication. Numerous lines of evidence from research on other virus systems showed that the *cis*-RNA elements are indispensable for RdRp-mediated viral RNA synthesis (You et al., 2004; Filomatori et al., 2006; Sun and Simon, 2006; Hu et al., 2007). Thus, it is possible that the identified *cis*-elements in pG_N_/G_C_ have a regulatory function. However, due to the lack of tospoviral infectious clones, it is difficult to test potential protein interactions with the RNA genome of TSWV *in vivo*. The majority of CCA protein molecules enter plant nucleus rapidly after their biosynthesis (Yakir et al., 2009), which increases the difficulty of investigating the function of CCA1 in cytoplasm.

Another hypothesis is that TSWV may have obtained these *cis*-acting elements from the host plants during viral evolution. There is evidence that RNA viruses may acquire host-derived sequences during reverse transcription, illegitimate recombination with retrotransposons, and host genome integration-excision processes (Geuking et al., 2009). It is possible that TSWV might have undergone a similar route to acquire sequences from the host. Moreover, TSWV uses a cap-snatching mechanism for transcription initiation, which may also help the acquisition of host sequences. After being integrated into the virus genome, these elements may or may not have significant impact on the biological activities of TSWV in nature. The observation that CBS, AuxRE, and transcription initiation sites are not present in all the known TSWV isolates may indicate that these elements may not be critical to the virus and may get lost in subsequent selection and mutation events.

To our knowledge, this is the first report that the 5′ region of a viral RNA genome contains motifs suggestive of promoter elements. These elements were found to be light- and hormone-responsive when expressed as cDNA in a plant. It remains to be seen if these elements have a role in the virus lifecycle. Overall, we provide new clues on the origin and evolution of a bunyavirid genome sequence, and serves as a starting point to dissect the functions of end-genome or non-translational regions in RNA viruses.

## Data Availability

All datasets for this study are included in the manuscript and the Supplementary Files.

## Author Contributions

All authors designed the project, wrote the manuscript, read and approved the final manuscript. YZ and HP performed the experiments and analyzed the data. HRP and MMN guided the experimental work.

## Conflict of Interest Statement

The authors declare that the research was conducted in the absence of any commercial or financial relationships that could be construed as a potential conflict of interest.

## Footnotes

1 http://arabidopsis.med.ohio-state.edu/

## Supplementary Material

FIGURE S1 The *cis*-regulatory elements identified from the 5′-upstream 500-bp regions (including both non-translational sequences and the 5′-terminal gene sequences) of four *Tomato spotted wilt tospovirus* (TSWV) genes (cDNAs): RdRp, NSm, NSs, and N. Their corresponding 500-bp fragments are named as **(A)** pRdRp, **(B)** pNSm, **(C)** pNSS, and **(D)** pN, respectively. The underlined sequences are untranslated regions. pN has putative transcription initiation sites and light-responsive transcription factor binding elements.

FIGURE S2 The promoter activity of *Cauliflower mosaic virus* (CaMV) 35S is not significantly affected by light or auxin. qRT-PCR assays showed no significant difference of *GUS* expression in four-day old p35S:*GUS*/*Col*-0 seedlings grown in dark, 80 μmol m^-2^ s^-1^ continuous white light, and white light plus 10 μM IAA, respectively. Two independent transgenic lines (#1 and #2) were used for each qRT-PCR assay. Three biological replicates were used for each data point. The error bar denotes SEM. Significance of difference was tested using unpaired Student’s *t*-test.
