# Supplementary figures and images for: Putative Auxin and Light Responsive Promoter Elements From the Tomato spotted wilt tospovirus Genome, When Expressed as cDNA, Are Functional in Arabidopsis
Source: Front Plant Sci. 2019 Jun 28;10:804. doi: 10.3389/fpls.2019.00804 (PMC6611158; doi:10.3389/fpls.2019.00804)

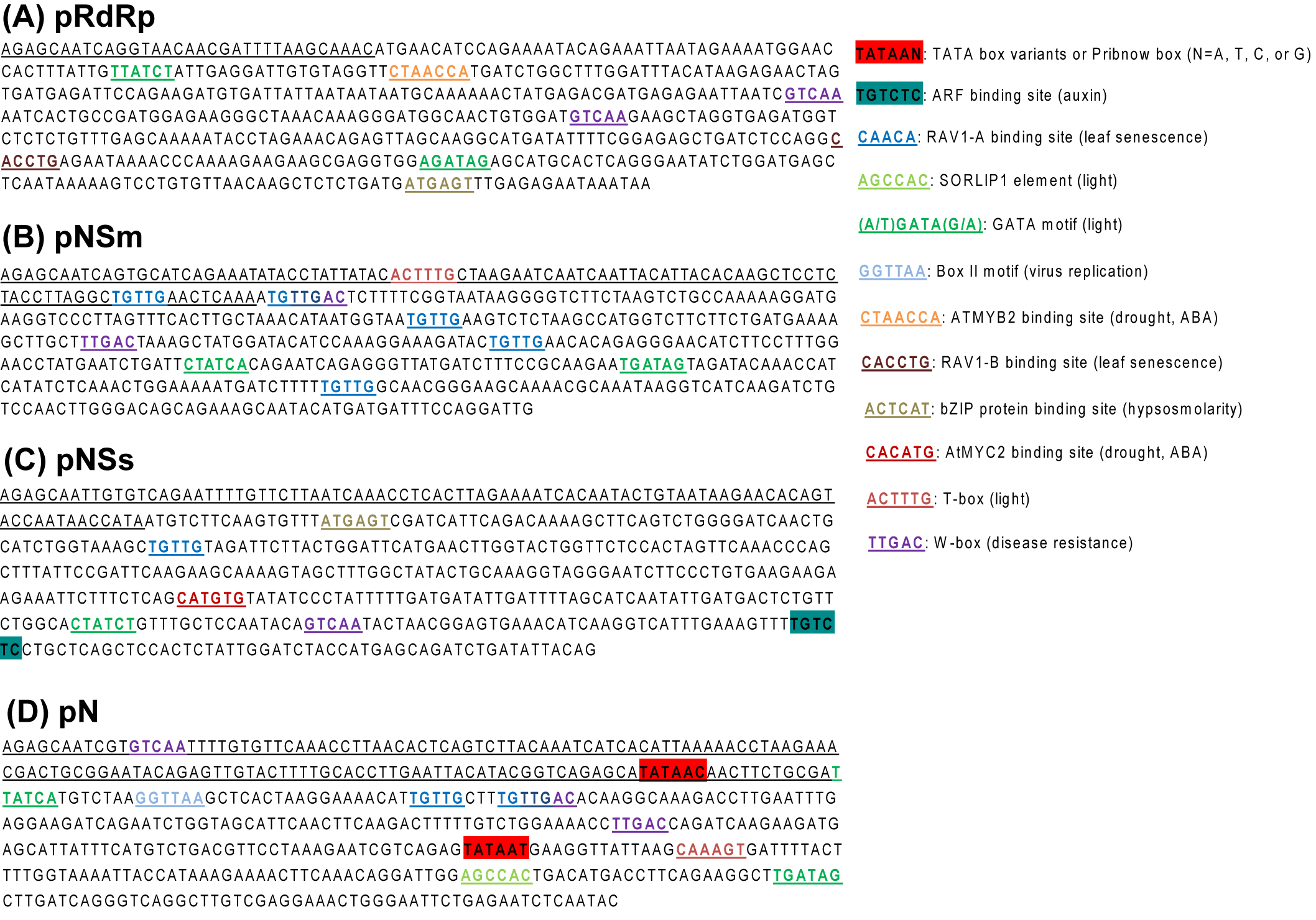

Supplement: FIGURE S1 — The cis-regulatory elements identified from the 5′-upstream 500-bp regions (including both non-translational sequences and the 5′-terminal gene sequences) of four Tomato spotted wilt tospovirus (TSWV) genes (cDNAs): RdRp, NSm, NSs, and N. Their corresponding 500-bp fragments are named as (A) pRdRp, (B) pNSm, (C) pNSS, and (D) pN, respectively. The underlined sequences are untranslated regions. pN has putative transcription initiation sites and light-responsive transcription factor binding elements. [file Image_1.TIF]

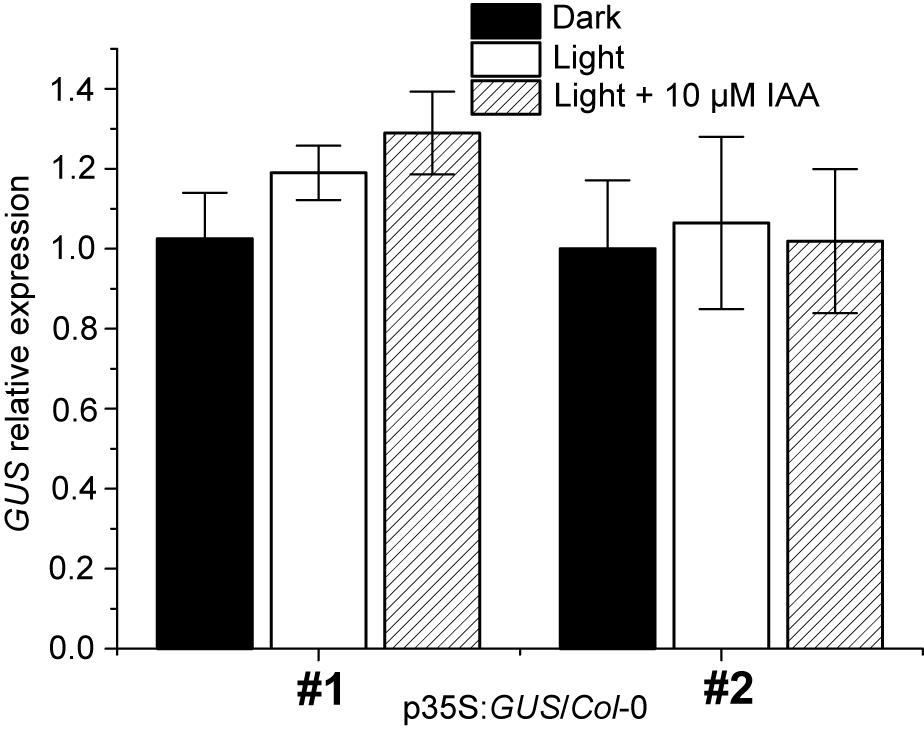

Supplement: FIGURE S2 — The promoter activity of Cauliflower mosaic virus (CaMV) 35S is not significantly affected by light or auxin. qRT-PCR assays showed no significant difference of GUS expression in four-day old p35S:GUS/Col-0 seedlings grown in dark, 80 μmol m-2 s-1 continuous white light, and white light plus 10 μM IAA, respectively. Two independent transgenic lines (#1 and #2) were used for each qRT-PCR assay. Three biological replicates were used for each data point. The error bar denotes SEM. Significance of difference was tested using unpaired Student’s t-test. [file Image_2.TIF]
